# Passive recruitment reach of a lifestyle management program to address obesity in the deep south during the COVID-19 pandemic

**DOI:** 10.3934/publichealth.2023010

**Published:** 2023-02-28

**Authors:** Jennifer L Lemacks, Laurie S Abbott, Cali Navarro, Stephanie McCoy, Tammy Greer, Sermin Aras, Michael B Madson, Jacqueline Reese-Smith, Chelsey Lawrick, June Gipson, Byron K Buck, Marcus Johnson

**Affiliations:** 1 Telenutrition Center, Mississippi INBRE Community Engagement and Training Core, The University of Southern Mississippi, Hattiesburg, Mississippi; 2 School of Health Professions, College of Nursing and Health Professions, The University of Southern Mississippi, Hattiesburg, Mississippi; 3 College of Nursing, Florida State University, Tallahassee, Florida; 4 School of Kinesiology and Nutrition, The University of Southern Mississippi, Hattiesburg, Mississippi; 5 School of Psychology, Mississippi INBRE Community Engagement and Training Core, The University of Southern Mississippi, Hattiesburg, Mississippi; 6 My Brother's Keeper, Inc., Mississippi INBRE Community Engagement and Training Core, Jackson, Mississippi

**Keywords:** obesity, minority health, nutrition, physical activity, recruitment, lifestyle

## Abstract

Obesity is a significant public health concern, especially in the Deep South and in Mississippi where prevalence is among the worst in the nation paired, with other poor health outcomes and socioeconomic conditions. Lifestyle management programs that address modifiable risk factors, such as nutrition and physical activity, can be effective mitigation strategies to halt weight accumulation patterns and ameliorate metabolic risk factors for some populations. However, there is limited evidence regarding the implementation of effective practice models to address obesity risk in underserved and underrepresented populations, such as African Americans, and people in the stage of earlier adulthood. Furthermore, there is growing evidence supporting the impact of the COVID-19 pandemic on lifestyle management programs that should be considered in these populations. The purpose of this manuscript was to describe the development and telehealth implementation of a weight management program during the COVID-19 pandemic and provide a preliminary examination of recruitment strategies and baseline characteristics for enrolled participants. Passive recruitment (social media, web, email, and other media advertisements) resulted in 157 screening initiations, and 79 of those participants met the study inclusion criteria. Further, of the 79 eligible participants, 38 completed all study enrollment requirements and presented with metabolic abnormalities. The study findings add to the emerging body of evidence for how the pandemic may have impacted lifestyle management programs and is representative of an understudied and underrepresented population.

## 1. Introduction

Obesity remains a significant public health concern in the United States (US). The age-adjusted prevalence of obesity is 42.4% in the US for adults with no significant difference between men and women or age groups [1]. In response to this growing epidemic, the US Preventive Services Task Force (USPSTF) recommended referral of obese individuals, those with a body mass index (BMI) of 30 kilograms per meter squared (kg/m^2^) or greater, to intensive, behavioral weight management programs [2]. Following this recommendation, the Center for Medicaid and Medicare Services (CMS) provided beneficiaries with obesity coverage for Intensive Behavioral Therapy (IBT), addressing nutrition and physical activity for weight management by primary care providers. Specifically, the benefit includes 14 to 15 brief, weekly face-to-face counseling sessions in the first 6 months, with additional monthly sessions (months 7–12) for those who lose more than 3 kg at 6 months [3]. IBT for obesity has been shown to be more successful than traditional weight-loss programs [4]. However, the time and expenses demanded of clinical settings and primary care providers may place too great a burden to be practical in a typical clinical setting, especially in areas with existing obesity disparities and underserved populations.

The research representing African American adults enrolled in behavioral interventions is very limited [5]. Previous research studies that included African American men indicated that lack of trust and poor relationships with healthcare providers diminished the acceptability of receiving potentially effective weight-loss programs in clinical settings [6]–[9]. Additionally, having positive role models and being in a familiar, relatable, and safe environment with health professionals that understand their priorities are ideals identified by African Americans as being important factors that contribute toward their overall well-being and quality of life [9],[10]. The overall goal of this study was to assess the implementation of an evidence-based intensive behavioral therapy program (with a motivational interviewing [MI] framework) in an outpatient clinical setting. Technological supplementations were utilized to address obesity and related chronic disease among younger adults (25 to 50 years of age), with an emphasis on African Americans and including other populations for comparison, using an adapted and comprehensive implementation fidelity model [11].

Following the onset of the coronavirus (COVID-19) pandemic in March 2020, non-pharmaceutical interventions, such as stay-at-home orders, were key strategies to limit the spread of the virus and reduce the overwhelming strain on healthcare systems. While these containment regulations aimed to reduce the spread of COVID-19, this also led to the shutdown of ancillary healthcare services, such as in-person IBT for obesity programs. To prevent the complete shutdown of the current study, the program was reconfigured for telehealth delivery. Accordingly, the purpose of this manuscript is to describe the intervention development and implementation of an IBT obesity program conducted via telehealth during the COVID-19 pandemic and provide a preliminary examination of recruitment strategies and baseline characteristics for enrolled participants.

## 2. Materials and methods

### 2.1. Study design and setting

Based on prior research with Mississippians who self-identify as African American, the program focused on weight loss in relation to disease risk reduction, instead of body ideals [11]. The pre-post intervention design included 20 sessions over 12 months with weekly visits in month one, biweekly in months two through six, and once a month in months seven through 12. Data collection time periods were at baseline and at the 6 and 12-month time points. The program was structured in alignment with the CMS IBT for Obesity benefits [12]. Health coaches who delivered the intervention received 16 hours of training in human subject research ethics, study design and protocol as well as motivational interviewing (MI) basic skills and participated in monthly “grand rounds” meetings to facilitate MI skill development and integration into practice and monitoring and feedback of MI skill. Health coaches utilized a facilitator guide, adapted from a prior Move & Eat 2 Live (ME2L) group-based curriculum [13], to deliver 20-minute intensive individual sessions that focused on setting and monitoring individualized diet and physical activity goals.

The ME2L intensive, individual (ME2L-II) format intervention was originally designed to test a mixed-methods delivery mode to alleviate the clinical burden that an intensive intervention can pose on physical staff and space resources by alternating face-to-face and telehealth delivery with only telehealth delivery in months eight through 12. With the onset of the COVID-19 pandemic, all sessions were delivered via telehealth and COVID-19 screening protocols were implemented prior to any face-to-face contact for data collection visits. Data collection visits occurred at the three major time periods (baseline, midpoint, and post) in an outpatient primary healthcare setting. All study protocols and procedures were approved by the University of Southern Mississippi Institutional Review Board (protocol 21–072). The trial has been registered with Clinical Trials.gov (NCT03855566).

### 2.2. Sample and recruitment

The inclusion criteria were individuals between 25 to 50 years old, residing in or able to commute to the Hattiesburg or Jackson, Mississippi areas, and had a BMI of 28 or greater. Exclusion criteria included contraindications for weight loss, cancer, chronic obstructive pulmonary disorder, emphysema, cystic fibrosis or any other major lung disease, liver or kidney dysfunction, end-stage renal disease, active hepatitis, celiac disease, colitis, or other gastrointestinal disorders or any other major health conditions. Other exclusion criteria were current pregnancy or within six months postpartum at baseline, heavy drinking, unintentional weight loss of more than 5% of body weight within the past six months, history of a heart attack or major heart surgery, or having been diagnosed with eating disorders (anorexia or bulimia nervosa), major depressive disorders, stroke, or severe psychological disorders that would interfere with diet and physical activity goals.

The initial participant recruitment plan included both passive and active recruitment efforts. Passive recruitment involved sending study announcements through patient portals, distributing fliers in public locations or housing developments, and solicitation notifications via email, social media, or other digital methods. Active recruitment efforts refer to outreach events throughout the community, whether at a brick-and-mortar location, through a community leader, or via a mobile clinic. Due to the COVID-19 pandemic, most recruitment methods were passive recruitment approaches (social media, emails, etc.). Potential participants, who were interested in the program, were contacted via email or by phone call and given a quick response code or weblink to access the study screener, program orientation, and provided consent with the inform consent module through a secure REDcap emulator webapp.

### 2.3. Instruments

Recruitment information was collected from the digital screener, orientation, and informed consent online module process that were all located inside the REDcap emulator webapp. Participants were asked how they heard about the ME2L program which included answer options such as church, healthcare provider/clinic, community event/health fair, social media, friend, family or another person, university/college campus, pharmacies like Walgreens or CVS, grocery or supermarket, gym or fitness center, nutrition or supplement store, seeing or receiving a flyer at an apartment complex.

The primary outcome for this study was weight which was collected in pounds and kilograms to the nearest tenth utilizing a Tanita Body Composition Analyzer MC-780U. Secondary clinical measures included BMI (kg/m^2^), girth circumferences (abdomen, hip and waist measurement to the nearest tenth of an inch using a Seca 201 nonflexible tape), body fat percentage, blood pressure, glucose level, and lipid panel analyses. Routine clinic procedures guided the collection of blood samples that were sent to a local laboratory for blood glucose and lipids analyses. The results were shared between participating clinics and academic entities through a data-sharing and protected health information release agreement.

The baseline survey, utilized for data collection, included 190 survey items that took approximately 45 minutes to complete. The baseline survey was accessed and delivered through the Bridge2U mobile web-application. Survey items included demographics, medical and weight history, and psychosocial and behavioral variables related to diet and physical activity. Dietary intake was assessed using the National Cancer Institute Dietary Screener Questionnaire [14],[15]. Computed dietary factors included fruits and vegetables (cups per day), fiber (grams per day), added sugar (teaspoons [tsp] per day), sugar from sweetened beverages (tsp per day), and red/processed meats (days consumed per week). Physical activity levels were determined using the International Physical Activity Questionnaire (IPAQ) short form [16], which has been validated (*a* = 76) in previous research [17].

### 2.4. Procedures

The digital screener module was a preliminary determination to study eligibility based on age, height, weight, and BMI. Informed consent was obtained for study participation. After participants agreed to be a part of the study, they would advance to the orientation module for study site location selection and to view a five-minute video describing the health program in full. As part of the standard informed consent process, participants were asked to authorize the use and disclosure of protected health information between the outpatient care clinics and research entities, and also certify/agree with the study's participant medical release statement. Once all criteria were met, the research team scheduled enrollment appointments with the participants' preferred clinical site locations. Participants were also sent a “Welcome” email message with instructions on how to register with the Bridge2U mobile web app to complete the baseline (or assessment) surveys. Once the web app registration was complete, a study identification code was generated, and each participant was given an assigned study ID number. For participants who were unable to register with the Bridge2U mobile web app, the research staff would assist them during their enrollment visit.

Enrollment visits were conducted at the clinical site location and included a free health/wellness screening program offered by the clinic. Clinic and research staff collected clinical biometric screenings at baseline, which included the following: anthropometric measurements (waist girth, high-hip girth, and hip/seat girth), blood pressure, glucose, and lipid panel analysis. During the enrollment visit, all measures were recorded and transferred into secure folders that were shared between clinic and research personnel for data entry into respective electronic systems. Once lipid panel results returned from the laboratory, lipid panel data was entered into the research portal for data collection. After enrollment data were collected from each participant, an introductory intervention session with a health coach was scheduled for the following week. The participants were advised to complete other baseline assessment surveys by the next session. All health coach sessions were scheduled after the enrollment visit for the remainder of the study duration. Participants received a $25 gift card for completing all baseline requirements, including clinical/biometric data collection during the enrollment visit and the completion of the baseline survey via the Bridge2U web app.

### 2.5. Data analysis

Descriptive statistics (averages for quantitative variables and percentages for qualitative variables) were computed using IBM SPSS Statistics 25 to describe the results of recruitment efforts, attrition, sociodemographic characteristics of study participants, and baseline anthropometric, clinical, dietary, and physical activity data.

## 3. Results

**Figure 1.**
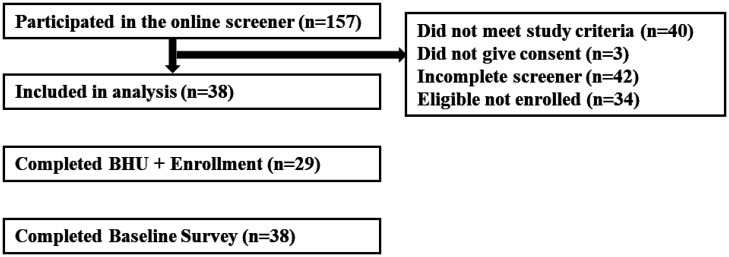
Recruitment, eligibility, and enrollment numbers for ME2L program.

A total of 157 individuals participated in the online screener, and of those, 119 people were excluded (Figure 1). Some individuals (n = 40) did not meet eligibility criteria because they had a calculated BMI less than 28.0 (n = 21), were younger than 25 years old (n = 13), were older than 50 years of age (n = 5), or had a contraindication for weight loss (n = 1). Some individuals (n = 42) did not complete the screener module, and a few others (n = 3) did not provide informed consent. There were 34 individuals who were eligible to participate but did not enroll in the study. A majority of individuals (71%, n = 23) either did not respond to scheduling an enrollment appointment or did not attend their scheduled enrollment appointment.

Participants mean age (± standard deviation) was 38.2 (6.94). Participants (n = 38) identified being recruited via healthcare clinics (24%; n = 9), churches (24%; n = 9), family/friends (21%; n = 8), social media (16%; n = 6), and flyers placed in apartment complexes (16%; n = 6). Data were collected and analyzed to describe the sociodemographic characteristics, medical history, and weight-related behaviors of the participating sample (Table 1). Most participants were female (79%) and self-identified as African American (82%). Most participants were female (79%) and self-identified as African American (82%). Some study participants reported having high blood pressure (37%), diabetes (10%), and many reported a family history of obesity (94%), high blood pressure (84%), diabetes (63%), reflux (58%) and high cholesterol (47%). Regarding weight-related behaviors, exercising (21%) or recording food intake (16%) behaviors were reported less often than regular monitoring of their weight and eating breakfast regularly (53%).

**Table 1.** Participant characteristics for ME2L enrolled participants at baseline (n = 38).

Categorical demographics	Category labels	% (n)
Age group	25–35 (young adults)	42 (16)
	36–50 (middle-aged adults)	58 (22)
Gender	Female	79 (30)
Race	White	13 (5)
	African American	84 (32)
	American Indian or Alaskan Native	3 (1)
Highest level of education	Some college but not a college degree	21 (8)
	A 2-year or vocational degree	16 (6)
	A 4-year college degree or higher	63 (24)
Individual current income level	I am currently unemployed	13 (5)
	$0 to $19,999	5 (2)
	$20,000 to $39,999	32 (12)
	≥ $40,000	49 (19)
Current marital status	Single	40 (15)
	Married	40 (15)
	Cohabitating	5 (2)
	Divorced	8 (3)
	Separated	2 (1)
Participant medical history		
High blood pressure		37 (14)
Diabetes		10 (4)
Heart attack or Stroke		0 (0)
High cholesterol		24 (9)
Family medical history		
High blood pressure		84 (32)
Diabetes		63 (24)
Heart attack or stroke		24 (9)
High cholesterol		47 (18)
Weight-related behaviors		
Check weight using a scale at least 4–5 times a week		40 (15)
Eat breakfast at least 4–5 times per week		53 (20)
Exercise at least 4–5 times per week		21 (8)
Record your food intake at least 4–5 times per week		16 (6)

Clinical measures for participants enrolled in the ME2L program were collected at baseline (Table 2). Although 38 participants were enrolled to participate in the study, clinical enrollment data was not collected from nine participants due to the clinic closures related to the pandemic. Of the 29 participants enrolled prior to clinic closures, most had a BMI classification of obese (93%; n = 27) with more women classified as obese (96%; n = 22) compared to men (83%; n = 5). Most participants (68%; n = 19) were considered stage 1 hypertensive with systolic pressures ranging from 130 to 139 mmHg or diastolic pressures ranging from 80 to 89 mmHg. Some participants had elevated triglyceride levels (24%; n = 6), elevated total cholesterol levels (31%; n = 8), elevated LDL levels (68%; n = 17), or low HDL levels (23%; n = 6). Most participants (91%; n = 20) had non-fasting glucose levels below 140 mg/dL, considered in the normal range.

**Table 2.** Clinical measures for ME2L enrolled participants at baseline.

Project	Overall		Male		Female	
	n	Mean (SD)	n	Mean (SD)	n	Mean (SD)
Anthropometrics^a^						
Weight (lbs)	29	219.4 (43.6)	6	238.3 (48.8)	23	214.3 (41.8)
BMI (Kg/m²)	29	36.38 (5.58)	6	34.22 (3.75)	23	36.89 (5.90)
Abdomen (inches)	29	41.45 (5.50)	6	43.49 (3.59)	23	40.89 (5.83)
Waist (inches)	29	43.01 (5.60)	6	44.09 (4.76)	23	42.83 (5.87)
Hip (inches)	29	46.40 (4.61)	6	45.50 (4.04)	23	46.63 (4.81)
Fat Percentage	29	40.26 (6.39)	6	31.60 (3.44)	23	42.51 (4.85)
Clinical^b^						
Systolic blood pressure (mmHg)	28	131.5 (12.9)	6	135.3 (12.6)	22	130.5 (13.4)
Diastolic blood pressure (mmHg)	28	84.46 (9.77)	6	85.84 (6.68)	22	84.32 (10.6)
Lipid triglycerides (mg/dL)	25	114.4 (57.5)	6	150.3 (73.8)	19	103.1 (48.3)
Total cholesterol (mg/dL)	26	189.0 (43.6)	6	182.7 (30.9)	20	190.9 (47.0)
HDL cholesterol (mg/dL)	26	51.77 (12.9)	6	45.50 (12.2)	20	53.65 (12.7)
LDL cholesterol (mg/dL)	25	113.7 (36.8)	6	110.2 (25.5)	19	114.8 (40.3)
Glucose (mg/dL)	22	118.3 (50.5)	5	98.80 (19.1)	17	124.0 (55.7)

^a^Note: A total of 29 were included in the analysis because nine were enrolled during the COVID-19 pandemic period when clinics were closed. ^b^Note: Sample sizes vary due to value (s) not available from the clinic.

Data regarding dietary intake and physical activity behaviors were also collected at baseline (Table 3). For dietary intake, the participants (n = 38) averaged less than a cup of fruit (*M* = 0.73, *SD* = 0.25) and more than one cup of vegetables (*M* = 1.57, *SD* = 0.23) per day. The average numbers of daily grams of fiber (*M* = 14.6, *SD* = 2.29), teaspoons of added sugar (*M* = 18.3, *SD* = 8.89) and sugar from sweetened beverages (*M* = 9.7, *SD* = 7.72) were computed as were the average number of times red (*M* = 0.46, *SD* = 0.46) and processed meats (*M* = 0.33, *SD* = 0.43) were consumed each day. For physical activity, the majority of participants (n = 29) were inactive (n = 13; 34%) or minimally active (n = 10; 26%).

**Table 3.** Diet and physical activity data for me2l enrolled participants at baseline.

Category	Mean (SD)
Dietary intake (n = 38)	
Fruits, cups/d	0.73 (0.25)
Vegetables, cups/d	1.57 (0.23)
Fruit and vegetables, cups/d	1.87 (0.42)
Fiber, grams/d	14.6 (2.29)
Added sugars, tsp/d	18.3 (8.89)
Sugar from sweetened beverages, tsp/d	9.7 (7.72)
Red meat, times/d	0.42 (0.46)
Processed meat, times/d	0.33 (0.43)
Physical activity (n = 29)	n (%)
Inactive (<600 MET-min/week)	13 (34)
Minimally active (600–1500 MET-min/week)	10(26)
Highly active (>1500 MET-min/week)	6 (16)

## 4. Discussion

This study provides information about the development and implementation of an IBT program targeting obesity in a southern, underserved population during the COVID-19 pandemic, including the recruitment and intervention delivery strategies that were employed. Our results showed that a much greater number of eligible people were engaged to at least initiate the screener, orientation and informed consent module but did not participate in the study compared to the number of participants who completed all of these modules and were enrolled. This finding suggests that although people were initially interested in finding out about the study, they were hesitant in completing the preliminary steps and committing to study participation and its related activities. Previous research has shown that while passive methods are more cost-effective than active recruitment methods, passive methods yield a lower percentage of enrolled participants [18]–[20], and the results of the current study supported this viewpoint. However, a positive observation was that passive recruitment did not hamper the enrollment of racially and ethnically diverse participants in this study as most (84%) self-identified as African American. The passive recruitment methods used during this study resulted in more enrolled females than males and accounted for nearly equal representation between younger and middle-aged adult groups. However, some difficulties arose when attempting to engage males in the health intervention, an issue that could be related to factors outside and possibly inside the recruitment modality and process. Previous research has reported that males, especially minority males, are difficult to recruit and retain in health interventions targeting weight loss and dietary changes [21],[22]. However, the prevalence of obesity in the United States among both males and females [1] underscores the need to address this public health problem and potentially prevent chronic diseases associated with obesity such as cardiovascular disease, diabetes, and cancer [23].

The findings of this study advance the literature on an underserved and underrepresented population regarding baseline anthropometric and clinical data such as BMI, plasma cholesterol and glucose levels, and blood pressure readings. It is worth noting that the criteria for inclusion in this study comprised having a BMI of 28 or higher, but the study results showed that the mean BMI value for the overall sample was much higher than the minimum value for inclusion and consistent for both males and females enrolled in the study. This finding provides a glimpse of the magnitude of the obesity problem in the southern United States that contributes toward health disparities and higher morbidity and mortality associated with chronic diseases prevalent in this region [23]. Although the non-fasting blood glucose levels were within normal ranges at baseline, the study included young to middle-aged adults who may yet experience metabolic changes with age, especially since obesity is a significant contributory risk factor of both prediabetes and diabetes, especially in minority populations [23]. Most participants had elevated LDL levels, and approximately a quarter had elevated triglyceride and low HDL levels. Further, the results for both systolic and diastolic blood pressure were above optimal ranges indicating that the participants had not yet been diagnosed with hypertension or were not adequately managing their previously diagnosed hypertension through lifestyle recommendations and/or treatment modalities daily. This highlights the importance of measuring blood pressure and other clinical parameters in lifestyle programs and providing information and/or referral to reduce complications such as stroke, heart attack, and kidney problems. However, measuring accurate and reliable clinical parameters was challenging following the clinical care and research encounter closures that occurred because of the COVID-19 pandemic. The research methods for this study were subsequently pivoted to a telehealth modality, but because of the shutdown that occurred during the pandemic, we were unable to obtain clinical measures for nine participants. The consequences of such missed opportunities can have implications for participants and potentially affect both health and research outcomes by limiting the implications of study conclusions.

The study results regarding dietary intake and physical activity behavioral risk factors suggest that improvements in preventive health services are needed to move the needle toward promoting healthier lifestyles and reducing disparities among underserved southern groups. For dietary intake, the participants generally consumed fewer than recommended servings of fruits, vegetables, and fiber each day as well as excessive amounts of sugar that was added to foods and sweetened beverages. Dietary guidelines recommend that adults consume 2.5 cups of vegetables and 2 cups of fruit each day and limit sugar intake [24]. Regarding physical activity, most of the participants were either inactive or minimally active. Most of the study participants reported that they had a family history of diabetes and hypertension. Although family history is a nonmodifiable risk factor, making even small strides in modifiable risk factors such as eating healthier and being more active can potentially reduce their risk for a future chronic disease diagnosis and impact future generations through the epigenome [25]. It is well established that increasing produce and fiber intake, reducing sugar consumption, and being physically active will usually facilitate weight loss and reduce the risk for obesity and other chronic diseases. Further research is needed to establish effective practice models for motivating and promoting lifestyle behavior changes, especially to meet the needs of underserved and underrepresented groups in the Deep South due to unique socioeconomic and geographic considerations.

While our results are reflective of the telehealth implementation of the IBT for obesity, which was initially designed for delivery in a primary care setting, our results could also be reflective of the pandemic period and not the recruitment modalities themselves. Although research has shown passive recruitment methods to be half as effective as active methods for lifestyle intervention enrollment pre-pandemic [18], it is plausible that participants were hesitant to enroll in the intervention because of the uncertainties involving the pandemic rather than the passive recruitment modalities. Although more research is needed, there is some evidence to suggest that lifestyle management programs aimed at weight loss and delivered during the pandemic period have not yielded desired and expected outcomes [26]. Others have reported higher BMI is associated with lower physical activity and overeating during lockdown periods [27]. Nuss et al. [28] were afforded the opportunity to examine group differences in 102 versus 90 families that participated in a 10-week family-based, child weight management intervention via a blended (virtual and face-to-face) delivery before the pandemic or an all-virtual delivery during the pandemic, respectively. Both delivery modes were viewed as equally effective based on post-intervention changes in lifestyle factors in sample distribution of mostly white families/children (45.8%) followed by 6.3% indigenous, 12.0% Asian (South Asian, West Asian, Chinese, and Southeast Asian), and 7.3% black or Latin American identified residents of British Columbia. A similar study [29] comparing groups of children/adolescents enrolled in a weight management program in Italy before (n = 80) and during the pandemic (n = 65) reported no differences in drop-out rates but did show worsened lifestyle factors in the pandemic group, which was attributed to decreased effectiveness of the intervention. Another study [30] representing predominantly White, non-Hispanic/Latino adults in Florida saw no decreased effectiveness in weight loss of a 16-week intervention based on the diabetes prevention program and delivered virtually during the pandemic; the sample had higher education levels (68.8% with at least a college degree and another 13.1% with an associate degree) and more than 75% of participants reported incomes greater than $50,000. Results are mixed, likely due to the vast cultural, socioeconomic, environmental, and geographical differences across study populations, which further highlights the need for research to represent the population included in this study.

Although the study had strengths regarding the methods, instruments, and ease of transition to a telehealth delivery format, there were some limitations. One limitation is that we were unable to ascertain how many people were reached and did not engage in the screener, orientation, and consent module. Data were not collected on individuals until they consented, so aside from the screener variables, it is unknown what other differences there might be between participants who were and were not enrolled. We were also unable to collect clinical measures on some participants because of clinic closures and protections placed by the institutional review board regarding face-to-face contact with research participants during the pandemic. Another limitation was that the participants were representative of a southern population and may not be generalized to other populations. This study also has implications for future research. For example, there is a need to understand factors that facilitate participant engagement and participation in web-based modalities. Future studies should determine best practices for study retention methods for African American and other underrepresented groups participating in weight loss and dietary health interventions).

## 5. Conclusions

This study has implications for research and practice and provides greater insight into passive recruitment outcomes, particularly among African Americans and other underserved populations with limited resources. This study also provides some insight into how lifestyle management programs and interventions were able to pivot to telehealth delivery and may have been impacted during the pandemic period. Further investigation is required to explore program attrition as well as other outcome impacts as a result of telehealth delivery and the pandemic.
